# Control of Brain Activity in hMT+/V5 at Three Response Levels Using fMRI-Based Neurofeedback/BCI

**DOI:** 10.1371/journal.pone.0155961

**Published:** 2016-05-23

**Authors:** Teresa Sousa, Bruno Direito, João Lima, Carlos Ferreira, Urbano Nunes, Miguel Castelo-Branco

**Affiliations:** 1 Institute for Biomedical Imaging and Life Sciences (CNC.IBILI), Faculty of Medicine, University of Coimbra, Coimbra, Portugal; 2 Institute of Nuclear Sciences Applied to Health (ICNAS), University of Coimbra, Coimbra, Portugal; 3 Institute of Systems and Robotics (ISR-UC), Department of Electrical and Computer Engineering, University of Coimbra, Coimbra, Portugal; Laureate Institute for Brain Research and The University of Oklahoma, UNITED STATES

## Abstract

A major challenge in brain-computer interface (BCI) research is to increase the number of command classes and levels of control. BCI studies often use binary control level approaches (level 0 and 1 of brain activation for each class of control). Different classes may often be achieved but not different levels of activation for the same class. The increase in the number of levels of control in BCI applications may allow for larger efficiency in neurofeedback applications. In this work we test the hypothesis whether more than two modulation levels can be achieved in a single brain region, the hMT+/V5 complex. Participants performed three distinct imagery tasks during neurofeedback training: imagery of a stationary dot, imagery of a dot with two opposing motions in the vertical axis and imagery of a dot with four opposing motions in vertical or horizontal axes (imagery of 2 or 4 motion directions). The larger the number of motion alternations, the higher the expected hMT+/V5 response. A substantial number (17 of 20) of participants achieved successful binary level of control and 12 were able to reach even 3 significant levels of control within the same session, confirming the whole group effects at the individual level. With this simple approach we suggest that it is possible to design a parametric system of control based on activity modulation of a specific brain region with at least 3 different levels. Furthermore, we show that particular imagery task instructions, based on different number of motion alternations, provide feasible achievement of different control levels in BCI and/or neurofeedback applications.

## Introduction

Multivariate supervised learning methods combined with real-time functional magnetic resonance imaging (fMRI) have allowed for the development of approaches enabling decoding of brain states based on the blood-oxygenation-level-dependent (BOLD) signal [1]. The decoded brain states can be used as control signals for a brain-computer interface (BCI) and/or to provide neurofeedback (NF) to the participant [2–4].

NF systems represent a BCI variant which allow training of voluntary regulation of brain activity, by feeding back information based on participants’ brain activity [5]. It is known that healthy participants can learn to self-regulate their local BOLD response with the help of real-time fMRI-based NF [6–14]. Specific behavioral effects have been reported as a result of NF in line with the idea that it allows for useful self-regulation of neuronal activity [9–11,14]. Accordingly, the potential importance of NF for learning self-regulation goes beyond what may be achieved by simple behavioral approaches based on the sole use of conscious cognitive strategies [8,9,15]. Clinical results of real-time fMRI-based NF studies focused at behavioral modulation do support its role as a novel non-invasive treatment tool for neurological and psychiatric disorders [15–20].

The correspondence between distinct brain activity patterns and particular perceptual states are key factors in determining whether cognitive states can be decoded. One typical case of such separation is given when different cognitive states/commands are encoded in spatially distinct locations of the brain [21]. In this case, BCI/NF studies are usually implemented using binary control level approaches. In other words, level 0 and 1 of brain activation are tested for a particular command. If multiple classes of control are aimed at, then multiple brain regions are needed to encompass several commands using this approach. Accordingly, a few studies have tested multiple classes of control based on a variety of mental tasks, but critically, they recruited distinct brain areas for different commands, and not the same brain region [22,23], or took advantage of different spatio-temporal aspects of the BOLD signal [24]. Different classes of control could be achieved but not different levels of up-regulation in the same region. Yoo et al. [22] explored the possibility of using real-time fMRI to interpret the spatial distribution of brain activity as BCI commands. They asked participants to perform four different mental tasks (‘right hand motor imagery’, ‘left hand motor imagery’, ‘mental calculation’, and ‘inner speech’) that evoke differential brain activation in four distinct brain locations and were interpreted as four BCI commands (four classes of control, each with a binary up-regulation level). Lee et al. [23] applied the same strategy to control a robotic arm movement. BOLD signals originating from the hand motor areas during right or left hand motor imagery tasks were translated into horizontal or vertical robotic arm movement (two classes of control with one up-regulation level). More recently, Sorger et al. [24] proposed a spelling device based on fMRI, by exploiting spatiotemporal characteristics of hemodynamic responses, evoked by performing differently timed mental imagery tasks (again, multiple commands, but only one up-regulation level).

When aiming to use real-time fMRI-based training for practical applications, it is relevant to demonstrate that training can produce beyond binary levels of control which might be advantageous as compared to those that can be achieved using conventional strategies. Therefore, it is of potential interest to assess parametric BCI/NF (with more than one level of up-regulation) [25]. The increase of the number of levels of control in BCI applications would allow for more powerful NF applications (different levels of volitional neuromodulation, i.e. more control levels over the activity of one particular brain region).

In this study we tested different visual imagery tasks with real-time fMRI-based NF in order to modulate the activity level of the same specific brain region, the hMT+/V5 complex, to verify whether more activation levels could be achieved than the typical binary case. We hypothesized that imagery tasks involving different numbers of imagined motion alternations, would lead to different levels of brain activity.

Pattern-based decoding of fMRI signals can successfully predict the perception of low-level perceptual features. For example, the orientation [26] and direction [27] of a motion visual stimulus presented to an individual can be predicted by decoding spatially distributed patterns of signals from local regions in early visual cortex. Decoding these patterns in imagery tasks is rather complex and none of these approaches alone has been successfully used to yield different levels of activation in imagery-based NF. Therefore, we decided to train the modulation of activity in the motion sensitive hMT+/V5 complex by exploring the alternative combination of multiple motion features, and to test three distinct visual imagery tasks based on those combinations.

We were inspired by the notion that strong and reliable fMRI responses are produced by the alternation of distinct grating orientations [28] in primary visual cortex (V1) in humans. In the motion perception domain, conditions for which motion alternations occur more often lead to stronger responses due to a break in brain response adaptation and/or higher attentional deployment. We adapted this notion to design our approach by hypothesizing that a larger number of motion alternations during the imagery tasks would lead to higher activity levels in hMT+/V5 as compared to a lower number of imagined alternations. We tested two up-regulation tasks using visual imagery strategies, imagery of a dot with two opposing motions in the vertical axis (imagery of two motion directions) and imagery of a dot with four opposing motions in vertical or horizontal axes (imagery of four motion directions) and one additional condition of down-regulation through the imagery of a stationary dot (zero/no motion). Our hypothesis is that three distinct levels of hMT+/V5 activity can be achieved based on these three imagery tasks with different number of motion alternations.

The visual region hMT+/V5 was chosen, since it is a well-studied motion sensitive area, at the intersection of the occipital, temporal and parietal lobes, often specified as located at the occipito-temporo-parietal pit, and easily identified through functional visual localizers with robust motion selective responses including imagery [29–31]. Response sensitivity to three-dimensional structure from motion is also well characterized in this region and in accordance with its monkey MT homologue [32,33].

## Materials and Methods

### Ethics Statement

This work was approved by the Ethics Committee of the Faculty of Medicine of the University of Coimbra. Twenty human volunteers gave written informed consent to participate in the experiment. The study has been conducted according to the principles expressed in the Declaration of Helsinki.

### Participants

Twenty volunteers (five female and fifteen male, between 20 and 53 years old, mean age = 28, SD ± 6.8, all right handed) with normal or corrected-to normal vision participated in this study. None of them had a history of neurological, major medical, or psychiatric disorders.

### Experimental design

First, participants were given instructions about the tasks and overall experiment. These included an explanation of the nature of feedback and recommendations concerning the regulation strategy (requiring imagery of different levels of movement direction alternation). We also explained the presence of a short time delay between the image acquisition and the feedback (which corresponds to the hemodynamic delay plus the real-time processing time). Then an anatomical scan was acquired. Participants performed a functional localizer task designed to identify the region-of-interest (ROI) hMT+/V5 over the left and right regions involved in motion processing. This ROI served as the subsequent signal source for NF runs.

Four imagery runs, each lasting nine minutes, with three different imagery tasks (visual imagery of the three types of stimuli shown during the previous localizer run) performed according to auditory instructions, were acquired. First, a passive imagery run (i.e. control run without feedback) was performed. In the two subsequent neurofeedback runs, participants attempted the up-regulation (two motion imagery tasks) and down-regulation (static dot imagery task) of the selected ROI fMRI signals assisted by a real-time auditory feedback. Finally, participants tried self-regulation in the absence of feedback during the final transfer run. They were asked to close the eyes throughout the four imagery runs, to breathe steadily, and to remain as still as possible.

The experimental design is further detailed in the next subsections.

### fMRI data acquisition

Scanning was performed using a 3T Siemens Magnetom TimTrio scanner, at the Portuguese Brain Imaging Network Central Facilities, using a 12-channel head coil. For each participant, scanning included the acquisition of five BOLD contrast echo-planar imaging (EPI) fMRI runs—one hMT+/V5 localizer experiment, one visual motion imagery run, two NF runs, and the final transfer run.

The recorded functional images consisted of 33 slices (field of view (FOV): 256 x 256 mm^2^, voxel size 4.0 x 4.0 x 3.0 mm^3^, flip angle (FA): 90°) yielding a total coverage of the occipital and posterior temporal lobe. Repetition time (TR) was 2000 ms (echo time (TE): 30 ms). For each participant, 200 volumes were acquired for the localizer run and 275 volumes for each NF, imagery and transfer runs. The beginning of each run was synchronized with the acquisition of the fMRI volumes.

Each scanning session included the acquisition of a high-resolution magnetization-prepared rapid acquisition gradient echo (MPRAGE) sequence for co-registration of functional data (176 slices; TR: 2530 ms; TE: 3.42 ms; voxel size 1.0 x 1.0 x 1.0 mm^3^; FA: 7°; FOV: 256 x 256 mm^2^).

### Real-time fMRI data processing

The fMRI setup used for real-time data processing was based on Turbo-BrainVoyager 3.0 (Brain Innovation, Maastricht, The Netherlands) [34] as previously described in [2].

Data were analyzed with real-time with Turbo-BrainVoyager software performing online 3D motion detection and correction, and drift removal. The statistical analysis was based on a parametric general linear model (GLM) and event-related averaging.

The feedback level was computed based on the mean activation level of the ROI of each incoming acquisition volume, i.e. at each TR the feedback was calculated based on percentage of ROI mean signal change in relation to the last baseline (down-regulation task) period. The feedback value was given to the participant using auditory instructions to prevent visual contamination/noise leading to additional signals in hMT+/V5 induced by motion in the visual field. The experimenter quantitatively forwarded the changes in mean activation level of the ROI, at each 2 TR, to the participant thus translating the exact levels of the standard visual “thermometer” scale of the Turbo Brain Voyager software (from level 0 –no activation to level 5—max activation).

### Functional definition of the target ROI (localizer experiments using visual stimulation)

Studies based on both single and multiple dots [29,35,36] have shown that a moving dot task reliably activates the hMT+/V5 complex [31].

**Fig 1:**
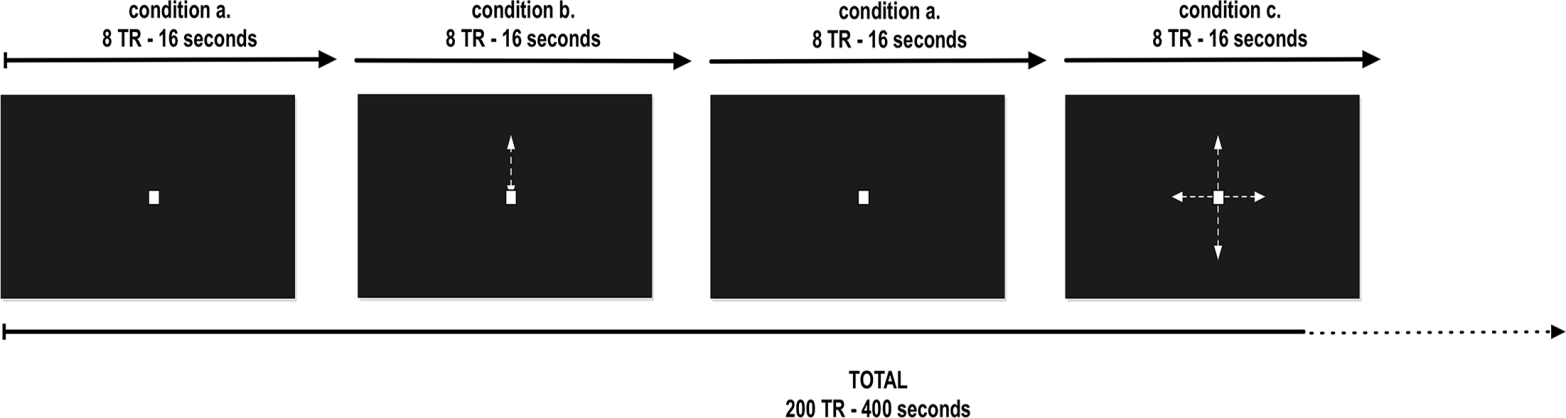
Functional localizer used to define the hMT+/V5 ROI. (A) Stationary dot, static condition used as baseline to the moving conditions depicted in (B) and (C). The arrows are here merely representing the dot motions used during the localizer. Each movement condition was repeated six times and randomly interleaved with the stationary dot condition. The duration of each block was 8 TR.

The NF target ROI, the hMT+/V5 complex, was determined using a functional localizer with 200 volumes. Participants were asked to fixate on a moving dot stimulus with two distinct movement conditions interleaved with a stationary dot. As described in Fig 1, the moving stimulus is a white dot either oscillating up and down along a vertical trajectory or with a back/forward movement according to a horizontal trajectory, against a black background. Three different conditions with different number of motion alternations were used: zero motion (stationary dot), a dot with two opposing motions (in the vertical axis) and a dot with four opposing motions (either horizontal and vertical axes). Sixteen seconds blocks of a moving dot (2.5 deg/s), were randomly interleaved with 16 seconds blocks of a stationary dot. The distance covered by the dot was 2 degrees of arc. The 2 blocks with movement were repeated 6 times, that resulted in a total of 25 blocks, 6 minutes and 40 seconds, 200 volumes. The point size was 0.5 x 0.5 cm^2^ and the stimulus was displayed at 44.5 cm (visual angle of the dot: 0.64 deg) from the subject at a screen of 24.1 x 18.2 cm^2^. Stimulus display was controlled by MATLAB (MathWorks) using the Psychophysics toolbox. The ROI was defined by selecting on the occipito-temporo-parietal pit all significantly activated voxels at *P* = 0.001 during the functional localizer run. The bilateral ROI size was not fixed, given that it was only dependent on the defined threshold.

### Neuromodulation runs (NF experiments using visual imagery)

After the anatomical and fMRI localizer scans, each subject performed four different real-time fMRI imagery runs: one control run of passive imagery, two NF runs and one control transfer run. Each run was composed by the three visual imagery tasks, matching the three visual stimulation conditions presented during the localizer run: imagery of a stationary dot, imagery of the dot with 2 opposing motions along the vertical axis and imagery of the dot with 4 opposing motions along either the horizontal or vertical axis. Instructions for each task/condition were provided as auditory cues and were coded as ‘A’, ‘B’ and ‘C’ respectively. Each run was composed of several up-regulation blocks (pseudorandomly presented dot movement imagery tasks, 6 repetitions of each task) interleaved by down-regulation blocks (imagery of a stationary dot). The duration of each block was 22 seconds. Each run lasted 9 minutes and 10 seconds (i.e. a total of 275 volumes per run).

The passive imagery (imagery without feedback) run was acquired as a control. With the exception of the feedback component, all the other parameters of this run are similar to the NF runs during which subjects performed different imagery tasks while trying to control the feedback value based on the activity of hMT+/V5. At the end of the NF training runs subjects were instructed to modulate their ROI activity using the same strategies, but now without feedback (transfer run). All runs were performed consecutively on the same session.

Subjects were not explicitly asked to try to achieve different levels of activation in the two different tasks of up-regulation (imagery of 2 or 4 motion directions). They were instructed to allocate similar effort (mere imagery) in the up-regulation conditions to achieve the highest level of activation possible. The goal was to achieve different activation levels depending on the used strategy (2 vs 4 motion directions) at a similar effort level. For the down-regulation condition (baseline) they were instructed to decrease as much as possible their ROI activity (imagery of stationary dot).

### Offline data analysis

The BrainVoyager QX 2.8.4 software version (Brain Innovation, Maastricht, The Netherlands) [34] was used for fMRI offline post-processing and analysis.

Pre-processing of the functional data included temporal high-pass filtering (GLM Fourier) with 2 cycles per run, space domain 3D spatial smoothing with a Gaussian filter of 4 mm, 3D motion correction with intra-session alignment and slice scan time correction with cubic spline interpolation. Functional data were co-registered to anatomical data per subject and subsequently normalized into Talairach coordinate space [37].

For each stimulus condition or imagery task presented to the subject, statistical maps were computed using a GLM [38]. The design matrix of the GLM was given by predictors encoding the stimulus conditions (localizer runs) and imagery task blocks (neuromodulation runs). The design matrixes were convolved with a double gamma hemodynamic response function in order to account for the hemodynamic delay and dispersion [39].

Statistical significant differences between each experimental motion condition and baseline (no motion condition), and between motion conditions were assessed using contrast (*t*) maps in each run. The contrasts *beta*, *t* and *P* values were analyzed after correction for serial correlations [38]. This correction was performed using a second-order autoregressive, AR(2), method [40]. A group random effect—RFX—analysis (with FDR correction for multiple comparisons) was performed to determine the ROI multi-subject cluster peak voxel resulting from the stimulation runs. Furthermore, supplementary whole-brain tests per imagery run were performed at the group level using fixed effects—FFX—analyses (with FDR correction for multiple comparisons) to list which other brain regions were recruited during the visual motion imagery. These analyses were performed separately for each run due to the fact that we include control and NF runs, and that inter-run variability is a recognized feature [41] (either due to learning and/or fatigue).

In order to verify if there was a group overall significant difference between the three different stimulation conditions (localizer visual stimulation experiments) and also between the three imagery tasks (imagery experiments organized in two groups of runs: imagery with feedback and the two types of imagery runs without feedback), we performed group analyses using linear trend tests and repeated measures ANOVA. These statistical analyses were based on percentage of signal change for each experimental condition extracted from the event-related averaging of each participant and using as baseline the average of every value of each pre-period (2 TR of the beginning of each condition) over the whole time-course. We also performed post-hoc tests to test between which conditions (static and 2 or 4 direction of motion imagery) occurred those differences. Furthermore, a two-way ANOVA was conducted to examine the effect of feedback on ROI response level. Chi-square tests and Pearson correlation were computed as well to analyze the putative influence of gender or age, respectively. Effects were only accepted as significant when *P* < 0.05 (corrected for multiple comparisons).

## Results

### hMT+/V5 localization

**Fig 2:**
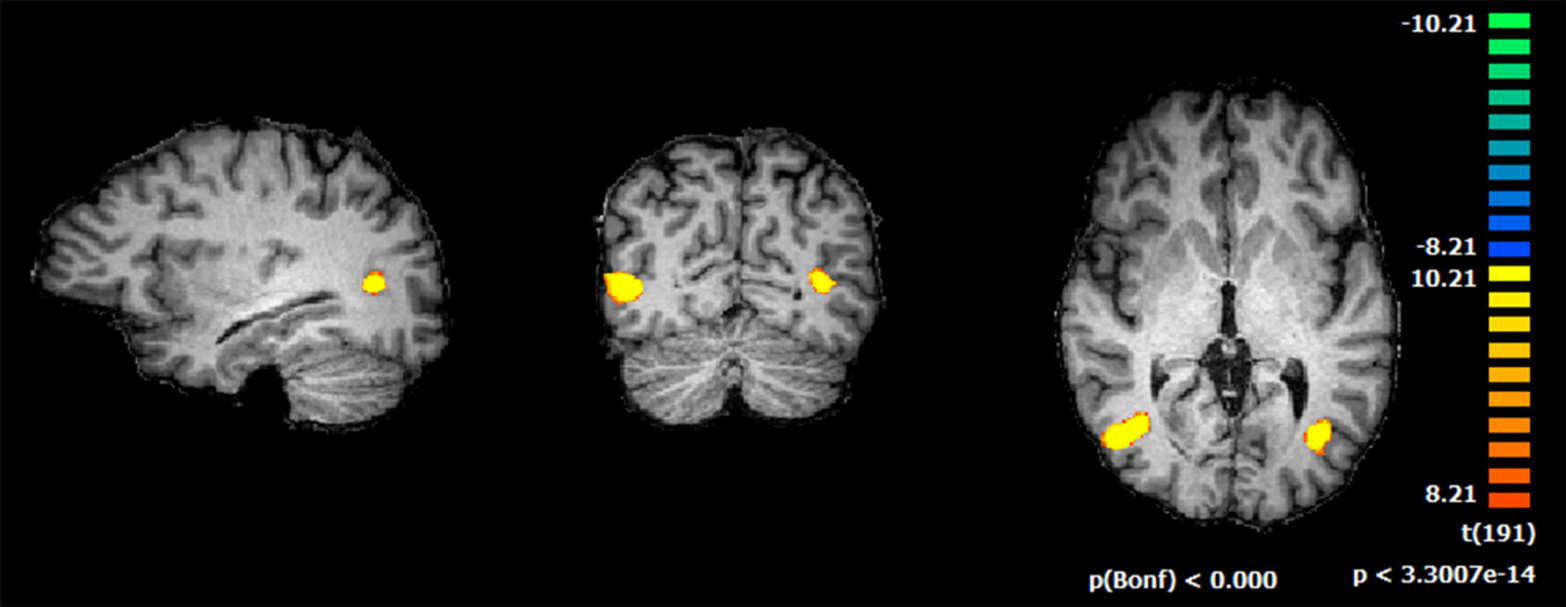
Example of hMT+/V5 identification using the defined localizer. GLM conjunction analysis (using the stringent criterion that all particular motion vs static BOLD signal contrasts had to be significant for a voxel to be considered positive) shows the resulting ROI: hMT+/V5. Regions are shown at the same statistical threshold level (*P* < 0.0001, Bonferroni corrected).

The GLM statistical maps resulting from the localizer run, (*P* < 0.05, Bonferroni corrected), revealed significant activations in the hMT+/V5 complex for each participant motion contrast (Fig 2, example from one participant), consistent with prior reports of its localization [30,31,42]. The ROI multi-subject cluster peak voxel center coordinates at *P* = 0.001 (RFX, *q(FDR)* = 0.05), were as follows (*x*, *y*, *z*): left (-44, -69, 2) and right (44, -68, 2).

Activation maps calculated in the offline analysis matched and validated activations maps observed in real-time using Turbo-BrainVoyager. This region was used as a ROI from which BOLD responses were extracted for each of the subsequent experimental conditions.

**Fig 3:**
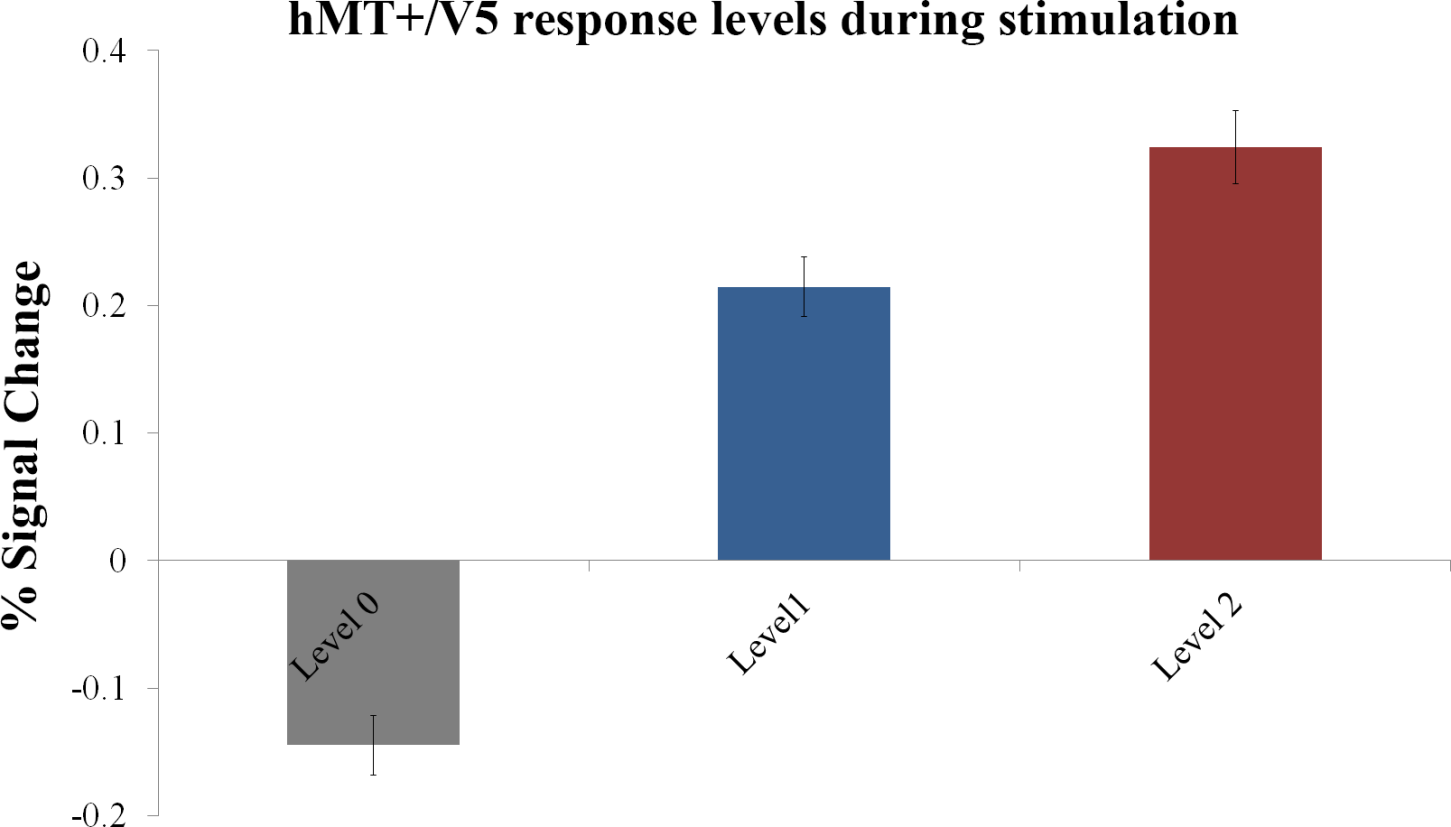
Mean BOLD activity within hMT+/V5 ROI during visual motion stimulation at different response levels. Group results for the localizer run, based on mean event-related BOLD response to the three stimulation conditions in the defined ROI per participant. Significant differences between all conditions were found at *P* < 0.05 (with Bonferroni correction for multiple comparisons). Values are presented as mean ± s.e.m. Level 0—stationary dot; level 1—dot with two opposing motions; level 2—dot with four opposing motions.

We predicted that different stimulation conditions with different quantity of motion alternation would lead to different levels of hMT+/V5 activity (for example 4 directions of motion lead to larger activity than 2 directions of motion). Accordingly, different ROI activation levels were observed as response to the different stimulation conditions (Fig 3). A repeated measures ANOVA determined that mean ROI activity differed significantly between stimulation conditions (*F* (1.52, 22.80) = 51.35, *P* < 0.0001). Post-hoc tests using the Bonferroni correction revealed that motion conditions evoked stronger hMT+/V5 activity than the zero motion condition (statistically significant at *P* < 0.0001). Furthermore, the two movement conditions evoked significantly different activity levels (*P* = 0.045), and larger activity was observed for the condition with 4 directions of motion.

### hMT+/V5 modulation: imagery based control of brain activity at 3 response levels

During the NF runs only three of the twenty participants were not able to modulate the hMT+/V5 activation. We considered that a participant was able to modulate the ROI activation when he/she showed an overall positive and statistically significant ROI response during at least one of the two up-regulation tasks in comparison to the down-regulation. From the remaining group, one participant achieved significant estimated effect (*beta* weight) for the 2 opposing motions imagery task, and sixteen participants were able to significantly increase (in relation to the down-regulation task) the defined ROI activity (*P* < 0.05) to both up-regulation tasks in at least one of the neurofeedback runs. Nevertheless, the contrast analysis between the ROI evoked responses during both up-regulation imagery strategies (with distinct number of imagined motion alternations), for each of these 16 participants, allowed us to verify that 12/20 participants showed not only a significant contrast between the evoked response during each of up-regulation task and the down-regulation task, but also a significant contrast between the responses evoked by the both up-regulation strategies (hereafter, we refer to this group of participants as the successful neuromodulators group).

**Fig 4:**
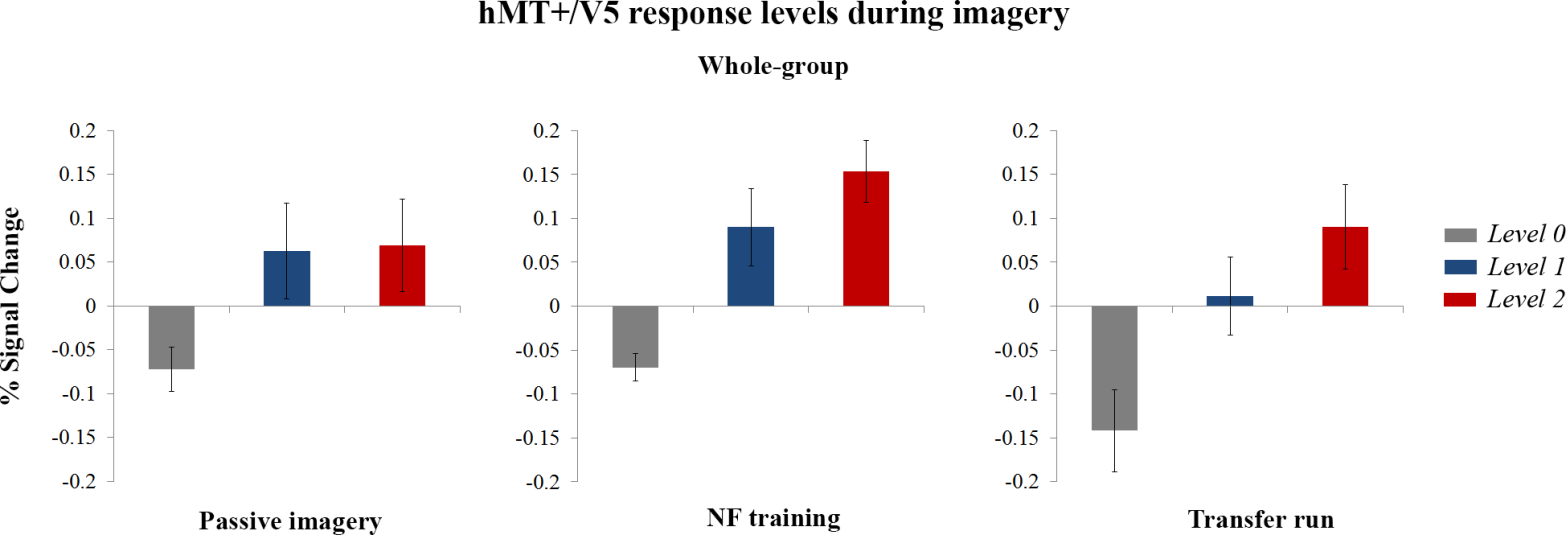
Mean BOLD activity within hMT+/V5 ROI during the neuromodulation runs with and without feedback. Group results (*N =* 20) based on mean event-related BOLD response to the three imagery tasks in the defined ROI per participant for neuromodulation runs with feedback and for neuromodulation runs without feedback (prior passive imagery and transfer runs). Values are presented as mean ± s.e.m. Three response levels were achieved depending on the applied imagery strategy (level 0: imagery of a stationary dot; level 1: imagery of a dot with two opposing motions; level 2: imagery of a dot with four opposing motions). A significant linear trend between response levels was found during the neurofeedback (*F* (1, 57) = 20.74, *P* < 0.0003) which persisted in the transfer run (*F* (1, 57) = 11.86, *P* = 0.003).

Globally, mean hMT+/V5 BOLD activity levels achieved during the up-regulation using the imagery of the dot with 4 opposing motion as strategy were higher than the mean activity resulting from the up-regulation using as strategy the imagery of the dot with 2 opposing motions (Fig 4). Fig 4 shows the mean group (*N* = 20) BOLD activity levels on the selected ROI for neuromodulation runs with and without feedback (separating the passive imagery and transfer runs) as result of the three imagery tasks. As illustrated, group results taking into account all the participants in the study show evidence for parametric neuromodulation (responses levels 0, 1 and 2) according the number of motion alternations involved in each visual motion imagery strategy. The comparison of the percentage of signal change achieved with distinct neuromodulation strategies using repeated measures ANOVA confirmed the main effect that mean ROI activity differed significantly between imagery tasks for all groups of runs: passive imagery (*F* (1.20, 22.79) = 6.29, *P* = 0.02), NF (*F* (1.50, 28.44) = 14.90, *P* < 0.0001) and transfer run (*F* (1.40, 26.68) = 11.19, *P* = 0.001). Furthermore, post-hoc comparisons between the 3 responses levels using Bonferroni correction revealed that differences between the response activity to the motion imagery strategies (level 1 and level 2) and the zero motion imagery (level 0) were significant for runs with (*P* = 0.02, *P* < 0.0001) and without feedback (passive imagery, *P* = 0.05 for both comparisons; transfer run, *P* = 0.03, *P* = 0.004). However, the differences between the level responses evoked by both up-regulation strategies were not significant, in particular passive imagery (*P* = 1.00) and NF (*P* = 0.124). Concerning the transfer run, a marginal effect was found (*P* = 0.06). In agreement with these findings, linear trend tests were not significant for the passive imagery run (*F* (1, 57) = 4.40, *P* = 0.12). Importantly, trend tests for level dependence were significant for the ROI response levels in the presence of neurofeedback (*F* (1, 57) = 20.74, *P* < 0.0003) and transfer run (*F* (1, 57) = 11.86, *P* = 0.003). A difference at the whole-group level of participants, concerning presence or absence of neurofeedback and its effect on the difference in response levels, was further confirmed by a two-way ANOVA (*F* (1, 19) = 5.22, *P* = 0.034).

For the successful neuromodulators group we found a main effect for all neuromodulation runs (passive imagery (*F* (1.38, 15.19) = 20.85, *P* < 0.0001), NF (*F* (2, 22) = 47.80, *P* < 0.0001) and transfer run (*F* (2, 22) = 22.46, *P* < 0.0001). Furthermore, the post-hoc tests with Bonferroni correction revealed that level 0 differed from level 1 and from level 2 for all runs (passive imagery, *P* = 0.004, *P* = 0.001; NF, *P* < 0.0001, *P* < 0.0001; transfer, *P* = 0.005, *P* < 0.0001). Differences between all responses levels were significant only the in the feedback runs (passive imagery, *P* = 0.97; NF, *P* = 0.002; transfer, *P* = 0.07). A linear trend was also found for all runs, with stronger effect sizes during NF training (passive imagery (*F* (1, 33) = 13.75, *P* = 0.003), NF (*F* (1, 33) = 51.23, *P* < 0.0003 and transfer run (*F* (1, 33) = 19.87, *P* = 0.0003).

In order to understand if age and gender had an influence in the ability of neuromodulation, we ran a chi-square test that shown no statistically significant association between gender and success of neuromodulation (*χ* (1) = 1.11, *P* = 0.292) and, a Pearson correlation was run to determine the relationship between individual's age and their performance in neuromodulation, which revealed no correlation between age and difference between maximal and minimal level of activity during the neuromodulation runs (*r* = 0.072, *N* = 20, *P* = 0.763).

The GLM results for group analyses per imagery run showed significant activations in the hMT+/V5 complex (FFX, *q(FDR)* = 0.05) during all performed neuromodulations runs. The recruited brain areas were similar in all runs (S1 Table). We observed the typical imagery parietofrontal network [43,44] and the hMT+/V5 complex.

## Discussion

We found evidence for the feasibility of BCI/Neurofeedback applications with different levels of modulation at the same brain location using the same strategy across participants. The possibility that parametric neuromodulation based on the same brain region and using the same strategy across participants is feasible had so far not been explored in the literature, although some alternatives have been suggested [25]. Previous studies have mainly tried to reach multiple command levels by analyzing not one but instead multiple regions, or by exploring particular spatio-temporal aspects of the BOLD signal [22–24].

Here, multiple visual motion imagery strategies which take advantage of differential evoked brain responses according to the number of imagined motion alternations, allowed achieving up to three distinct levels of hMT+/V5 activity modulation. The larger number (4) of motion alternations during imagery tasks evoked higher activity levels in hMT+/V5 as compared to imagery tasks with a lower number of motion alternations (2) and static imagery. These results can be explained by the fact that in human visual cortex frequent movement or orientation changes lead to break in adaptation and increased fMRI responses. Lower signal adaptation does indeed occur as compared to brain responses evoked by a movement with less alternation [28]. Our results are also consistent with the previous visual stimulation studies of Huk and Heeger [45] and Larsson et al. [46] where the visual stimulus with the highest rate of alternating evoked stronger brain activations. However, an alternative explanation is possible concerning the distinct levels of hMT+/V5 responses as a function of different imagery strategies. Accordingly, the different number of motion alternations may lead to distinct levels of attentional modulation which in turn impact on activity levels.

Trend analysis to the whole-group of participants revealed significant parametric neuromodulation during NF runs. Furthermore, there was no significant trend on response levels during passive imagery runs (control run without feedback previous to NF training). In contrast, a significant trend was found during the transfer run (control run after NF training). These results suggest that the provided feedback contributed for successful neuromodulation and learning during NF training. The comparison between runs with feedback and runs without feedback shown a significant feedback effect on the achieved response levels in agreement with the effects identified by group trend analyses.

The significant difference in level of modulation achieved with the use of auditory feedback based on the BOLD signal changes, suggests that the participants effectively use neurofeedback strategies. It is important to point out that auditory feedback was used to avoid interferences that would have been induced by spurious brain activations due to the visual feedback and that would perturb the signals evoked by the imagery task.

Future studies should address how many sessions are necessary to achieve stable performance. Binary neuromodulation (successful up-regulation during the two motion imagery tasks, and successful down-regulation during non-motion imagery) was often obtained (17/20 participants) and three levels of modulation were documented in a substantial number of subjects (12/20 participants). Future studies beyond proof of concept should establish whether training can stabilize NF performance. We suggest that three levels of control of hMT+/V5 activity are possible but also imply that they may require a higher level of focused attention and training than the binary case.

We have shown that the same strategy can be efficiently used by different participants to achieve modulation of hMT+/V5, and that the type of instruction can be useful in BCI and NF applications. Thus, this approach could be useful in the future to dampen the variability due to ROI and subject-by-subject strategy definition, and to allow more effective neuromodulation training or at least improved BCI control. We postulate that the proposed approach is promising for future NF applications and even more for BCI applications, because it provides a simple way to achieve three control levels with simple instructions.

## Conclusion

We demonstrated that both visual motion stimulation and imagery with different number of motion alternations levels lead to distinct activity levels in hMT+/V5, and this can be used effectively in a NF application. Up to three levels of volitional control of hMT+/V5 visual area by using real-time fMRI training could be achieved. The same imagery strategy was used by all participants, showing that the proposed novel methodology is of potential interest to implement in applications using level dependent parametric BCI and/or NF.

## Supporting Information

S1 Table Summary of whole-brain regions activated during visual motion imagery. Peak voxels according Talairach coordinates and the number of used voxels (FFX, *q(FDR)* = 0.05 using the contrast up-regulation tasks versus down-regulation task. Results presented per imagery run: passive imagery run (control run without feedback), imagery runs with feedback (neurofeedback 1 and 2, NF1 and NF2) and transfer run.
(DOCX)
