# Supplementary material for: Control of Brain Activity in hMT+/V5 at Three Response Levels Using fMRI-Based Neurofeedback/BCI
Source: PLoS One. 2016 May 23;11(5):e0155961. doi: 10.1371/journal.pone.0155961 (PMC4877110; doi:10.1371/journal.pone.0155961)
Supplement: S1 Table — Peak voxels according Talairach coordinates and the number of used voxels (FFX, q(FDR) = 0.05 using the contrast up-regulation tasks versus down-regulation task. Results presented per imagery run: passive imagery run (control run without feedback), imagery runs with feedback (neurofeedback 1 and 2, NF1 and NF2) and transfer run. (DOCX) [file pone.0155961.s001.docx]

|  |  | **Passive imagery** | | **NF1** | | **NF2** | | **Transfer run** | |
| --- | --- | --- | --- | --- | --- | --- | --- | --- | --- |
| **Brain region** | | Talairach coordinates (x, y, z) | Voxels | Talairach coordinates (x, y, z) | Voxels | Talairach coordinates (x, y, z) | Voxels | Talairach coordinates (x, y, z) | Voxels |
| hMT+/V5 | Left | -49, -60, -1 | 1248 | -44, -60, 0 | 5182 | -47, -60, -2 | 2482 | -48, -60, -1 | 1941 |
|  | Right | 46, -59, -4 | 690 | 48, -57, -4 | 2029 | 47, -61, -5 | 244 | 50, -58, -5 | 409 |
| Putamen | Left | -22, 1, 13 | 2335 | -22, 1, 8 | 4203 | -23, 2, 6 | 3398 | -23, 1, 9 | 4448 |
|  | Right | 21, 2 , 12 | 1982 | 23, 1, 11 | 3093 | 21, 3, 10 | 3177 | 21, 4, 8 | 3502 |
| Superior parietal lobule, Precuneus | Left | -21, -65, 48 | 3717 | -21, -68, 47 | 2734 | -21, -65, 46 | 3649 | -22, -66, 46 | 2891 |
|  | Right | 16, -67, 48 | 2666 | 15, -71, 46 | 2613 | 16, -66, 47 | 1957 | 14, -69, 42 | 2424 |
| Inferior parietal lobule | Left | -37, -43, 43 | 5930 | -34, -38, 37 | 4374 | -39, -41, 42 | 4674 | -38, -37, 38 | 4143 |
|  | Right | 38, -42, 42 | 5587 | 30, -46, 40 | 3573 | 34, -42, 44 | 2842 | 35, -43, 38 | 4018 |
| Precentral gyrus | Left | -49, -6, 40 | 3084 | -50, 1, 29 | 3767 | -51, 3, 22 | 3595 | -50, 2, 25 | 4532 |
|  | Right | 48, -4, 42 | 3430 | 52, 3, 27 | 3231 | 53, 3, 25 | 2945 | 50, 5, 24 | 4090 |
| Medial frontal gyrus | | 5, 12, 43 | 972 | -3, 4, 43 | 1745 | -5, 1, 46 | 1169 | -3, 10, 44 | 1178 |
